# Exploring the Effects of Virtual Reality on Pain Relief and Physical Mobility in Spa-Based Treatment

**DOI:** 10.3390/jcm14238510

**Published:** 2025-11-30

**Authors:** Alina Huseynli, Vojtěch Špet, Alena Lochmannová, Konstantin Novikov, Ladislav Špišák, Aleš Příhoda

**Affiliations:** 1 Institute of Spa and Balneology, Závodní 353/88, 36006 Karlovy Vary, Czech Republicspetvojt@cvut.cz (V.Š.);; 2 Faculty of Medicine in Pilsen, Charles University, Alej Svobody 1655/76, 32300 Pilsen, Czech Republic; 3 Department of Health Care and Population Protection, Faculty of Biomedical Engineering, Czech Technical University in Prague, nám. Sítná 3105, 27201 Kladno, Czech Republic; 4 VR Medical, Karlovarská 451/70, Severní Předměstí, 32300 Pilsen, Czech Republic

**Keywords:** musculoskeletal disorders, neurogenic disorders, pain management, physical mobility, spa-based rehabilitation, virtual reality

## Abstract

**Objectives**: The objective of this prospective, controlled observational study embedded in routine spa care was to evaluate the effectiveness of integrating immersive virtual reality (VR) into a three-week spa-based rehabilitation program to reduce pain and improve physical mobility in adults with chronic musculoskeletal or neurogenic disorders. **Methods**: In this study, fifty-five adults with chronic musculoskeletal or neurogenic disorders completed a three-week spa regimen combining natural therapies, physiotherapy and rehabilitation. Participants were allocated in a preference- and availability-based manner either to the VR-enhanced group (*n* = 37), which completed interactive 25 min VR sessions three times per week, or to the control group (*n* = 18) receiving standard care. Pain was assessed using a 100 mm Visual Analog Scale (VAS) and shoulder-related joint mobility by goniometry before and after the intervention. Wilcoxon signed-rank and Mann–Whitney U tests evaluated within- and between-group differences, with subgroup analyses according to disease duration (≤5 vs. >5 years). **Results**: Both groups achieved significant post-treatment reductions in VAS pain scores (*p* < 0.001). The VR group exhibited a greater median decrease in pain compared to controls (*p* = 0.048), with the largest effect among patients with disease duration ≤ 5 years (*p* = 0.024). Goniometric measurements demonstrated significant mobility improvements across all tested angles in the VR group (*p* < 0.001), while improvements in the control group were smaller. **Conclusions**: VR integrated into spa-based rehabilitation was associated with greater pain reduction and mobility gains than standard care. These preliminary, hypothesis-generating findings require confirmation in larger randomized trials with standardized protocols and long-term follow-up.

## 1. Introduction

The incorporation of virtual reality (VR) into healthcare has significantly transformed traditional therapeutic modalities [1,2], yet its utilization within spa-based rehabilitation remains insufficiently explored. Spa facilities, located in areas with favourable environmental and natural therapeutic resources, offer comprehensive rehabilitation and prevention programs that go beyond conventional medical treatments. Traditionally centered on holistic approaches, these therapies aim to alleviate pain, improve mobility, and enhance overall physical and psychological well-being [3]. The integration of VR into rehabilitation has shown considerable effectiveness in enhancing motor recovery [4]. VR applications, utilizing movement reproduction to translate physical actions into virtual environments, have proven effective for individuals with motor impairments [5]. Combining VR with traditional spa therapies further stimulates cognitive, sensory, and motor pathways, enhancing the therapeutic outcomes of conventional rehabilitation [6].

Chronic musculoskeletal and neurogenic disorders represent a significant public health challenge, markedly reducing individuals’ quality of life due to persistent pain, functional impairments, and restricted mobility [7,8,9]. These conditions may originate from identifiable pathological processes within the musculoskeletal or nervous system, but they frequently present as a clinical challenge when pain and functional limitations persist beyond the expected healing period, even in the absence of clear pathological findings in bones, joints, muscles, or neural structures. In such cases, traditional biomedical management approaches often fall short, as they may not adequately address the multifactorial nature of chronic pain, which involves not only physiological but also psychological and behavioral components [10]. To optimize therapeutic outcomes and enhance patient engagement, it is beneficial to complement intensive spa-based therapies with modern, technology-driven interventions [11]. Integrating conventional rehabilitation methods with innovative approaches such as VR provides a more comprehensive treatment strategy. VR’s immersive and interactive environments have been shown to improve motivation and adherence and to modulate pain perception by diverting attentional resources away from nociceptive stimuli [12,13,14,15,16,17]. Beyond pain relief, VR interventions may also support psychological well-being by enhancing focus, relaxation and emotional engagement in therapeutic activities [18,19]. Through repetitive, goal-directed tasks, VR-based exercises can facilitate motor learning and neuromuscular re-education, leading to improved motor function and coordination, as demonstrated in neurorehabilitation studies including post-stroke recovery and Parkinson’s disease management [4,20]. In the context of spa-based rehabilitation, which combines natural therapeutic resources, intensive supervised exercise and structured daily routines over several weeks [21], VR may therefore offer an additional way to address the complex interplay of physical, cognitive and psychosocial factors in chronic musculoskeletal and neurogenic disorders.

Although VR’s effectiveness in clinical rehabilitation is well-documented, its integration into spa-based programs remains limited. It is not yet clear whether adding VR to comprehensive residential spa programmes yields additional benefits beyond usual spa care. This prospective, controlled observational study therefore addressed three research questions. First, we examined whether VR-enhanced spa rehabilitation is associated with greater reductions in pain than standard spa rehabilitation. Second, we explored whether it is associated with greater improvements in upper-limb range of motion. Third, we assessed whether these effects differ according to disease duration. We hypothesised that patients receiving VR-enhanced spa rehabilitation would show larger reductions in VAS pain scores and greater gains in shoulder-related range of motion than those receiving spa rehabilitation without VR, particularly among participants with shorter disease duration.

## 2. Materials and Methods

This study was a prospective, controlled observational study embedded in routine spa care, which evaluated the effectiveness of immersive virtual reality during a three-week rehabilitation program to improve mobility and reduce pain. The spa-based rehabilitation program relied primarily on natural therapeutic resources and was complemented by movement therapy and standard physical-therapy procedures. These included treatments such as magnetotherapy, laser therapy, ultrasound therapy, distance magnetotherapy, thermotherapy (wraps), mineral baths, medical and reflex massages, and other therapeutic interventions. All procedures were individually prescribed on the basis of a physician’s medical assessment conducted at the initial examination, ensuring a tailored approach aligned with each patient’s specific health needs. This report adheres to the STROBE guidelines for observational studies conducted in routine care.

### 2.1. Ethical Approval

Ethical approval for the study was granted by the Ethical Committee of University Hospital Pilsen and Faculty of Medicine in Pilsen, Charles University, reference number 449/22.

### 2.2. Participants

As part of the study, patients with chronic musculoskeletal and neurogenic disorders, characterized by pain and mobility limitations, were monitored. These patients required targeted rehabilitation interventions to improve functional capacity and alleviate symptoms. In all cases, these were chronic and progressively debilitating conditions requiring comprehensive, interdisciplinary, and long-term care focused on promoting regeneration, pain management, and restoring functional independence in patients.

Participation in the study was subject to specific inclusion and exclusion criteria to ensure patient safety and the validity of outcomes. Eligible participants were adults aged 18 years and older whose medical condition allowed for active engagement in the prescribed rehabilitation program without contraindications to physical therapy interventions. Patients with acute inflammatory or degenerative conditions, severe neurological deficits impairing mobility, or cognitive impairments preventing adherence to the study protocol were excluded. The selection process was based on a physician’s assessment during the initial examination, ensuring an individualized approach to treatment. Enrolment in the study was entirely voluntary, and participants retained the right to withdraw at any stage without any impact on their ongoing therapeutic care.

The sample size was calculated for the model of observational study [22] while taking the internal therapeutic processes in the spa facilities into account. Because VR is used as a standard form of therapy in these facilities, the number of clients who do not undergo this form of therapy is much lower. Therefore, the exposed-to-control ratio was set at approximately 2:1 (k ≈ 2.0). Expected mean outcome in the control group *m*_0_ was set at 2 points on VAS with expected standard deviation of 2 points. Expected mean outcome in the experimental group *m*_1_ was determined by considering minimal clinically important difference, setting the value at 3.7 points. All estimates were based on the available evidence into the mean pain alleviation after therapy in chronic musculoskeletal diseases [23,24,25]. Prior to parameters, sufficient sample size for obtaining power of 1-β = 0.80 was calculated as 33 participants in experimental group and 17 in control group. To take the risk of participant dropping out of the research into account, final number was increased to 40 in experimental and 20 in control group.

### 2.3. Intervention

Participants were allocated to either the VR-enhanced pathway or standard care based on their preferences and scheduling availability within routine spa care. Individuals who declined VR were assigned to standard care. There was no random sequence generation and allocation was not concealed. Assignment was determined pragmatically by the attending spa physician and rehabilitation staff as part of routine clinical scheduling rather than by a prespecified randomization protocol.

The experimental group followed a specialized rehabilitation program integrating VR into their three-week treatment regimen, with VR sessions conducted three times per week, each lasting a maximum of 25 min. The VR intervention consisted of immersive therapeutic applications designed to resemble engaging, game-like experiences while, in reality, facilitating targeted motor and functional training. These applications included activities such as constellation recognition, fruit picking, fishing, and gardening, each requiring controlled movements aimed at improving coordination, motor function, and overall mobility. VR sessions were delivered using Oculus Quest 2 standalone head-mounted displays with integrated motion tracking and handheld controllers, together with a dedicated VR rehabilitation software system from VR Medical (Pilsen, Czech Republic) certified as a Class I medical device for clinical use.

The control group underwent a standard comprehensive spa rehabilitation program without VR, adhering to conventional therapeutic protocols. This study design allowed for a direct comparison of the effectiveness of VR-enhanced rehabilitation with traditional treatment approaches.

### 2.4. Instruments and Assessments

At the beginning of the rehabilitation program, baseline characteristics—including age, sex, occupational classification in terms of job role and work regime—were recorded for all participants in both study groups. Comprehensive functional assessments were conducted at the start and upon completion of the three-week spa treatment program, including goniometry to measure joint range of motion, the visual analog scale (VAS) for pain assessment, muscle strength testing and spinal mobility evaluation. Baseline and post-treatment assessments were performed by rehabilitation staff who were aware of participants’ group assignment, and neither participants nor assessors were blinded to the intervention.

The VAS is a widely used and straightforward tool for evaluating changes in pain intensity [26]. It consists of a 100 mm continuous line with verbal anchors at each end, typically ranging from “no pain” (0) to “worst imaginable pain” (100). The respondent marks a point on the line that best represents their pain intensity, and the score is determined by measuring the distance from the “no pain” anchor to the marked point in millimeters. The VAS is a single-item scale commonly used to assess current pain or pain experienced over the last 24 h. It is self-administered, with the respondent drawing a perpendicular line at their perceived pain level. To minimize response clustering, intermediate numerical values or descriptors are generally avoided [27].

All participants in the experimental group followed an identical VR-based rehabilitation program, ensuring standardized exposure to virtual therapeutic exercises. The VR applications were designed as therapeutic games facilitating targeted motor training for both upper and lower limbs, as well as core muscle activation. Before the initiation of the VR intervention, patients were thoroughly instructed by medical personnel on the study protocol and the safe use of VR headsets. Following this briefing, the headsets were applied, and participants were guided through an introductory session designed to familiarize them with the required exercises. The VR application then led them through a series of structured, engaging exercises specifically developed to promote mobility and coordination.

Before each VR session, the headset was individually fitted, and the virtual workspace was adjusted so that it matched the participant’s comfortable standing position and reach. A physiotherapist prepared an obstacle-free area and remained present throughout the VR exposure to ensure physical safety and to monitor the participant’s responses. In line with the study protocol, VR exposure was limited to a maximum of 25 min per session. Participants were informed about possible symptoms of cybersickness, including nausea, dizziness, headache and eye strain, and were instructed to close their eyes and notify the therapist immediately if such symptoms occurred. In such cases, the session was terminated and the headset removed. Patients with known epilepsy or severe visual disorders that could interfere with safe VR use, such as pronounced strabismus or marked uncorrected astigmatism, were not allocated to the VR group.

### 2.5. Data Analysis

Statistical analysis of the data was performed using IBM SPSS Statistics (Version 29.0.2.0 (20)), with the significance level α set at 5%. Effect size was interpreted based on the used method, with Cohen’s *d* (0.2–0.5 as small, 0.5–0.8 as medium and >0.8 as large effect) [28] and Partial eta squared (0.01–0.06 as small, 0.06–0.14 as medium and >0.14 as large effect) [29]. Baseline differences between the experimental and control groups were examined using Welch’s *t*-test including 90%CI compared with preset margin of equivalence [−1; 1]. Mann–Whitney U test was used as a non-parametric variant. For the evaluation of the continuous variable range of motion, the Shapiro–Wilk test was conducted to verify the assumption of normality, followed by Levene’s test for homogeneity of variances and Mauchly’s test of sphericity. If the null hypothesis of these tests was rejected, the use of appropriate nonparametric methods was considered. For the evaluation of pain using the VAS, as the variable represents an ordinal categorical measure, nonparametric methods were preferred, specifically the Wilcoxon signed-rank test, the Mann–Whitney U test for independent samples, and ordinal logistic regression analysis, although assumptions for parametric ANCOVA were evaluated as well, because of higher test power and relative robustness to minor assumptions deviation. Because allocation reflected participant preference and scheduling availability, we additionally fitted ordinal logistic regression models adjusted for age, gender, disease duration, occupation type, and physical activity.

## 3. Results

As for the attrition rate, a total of 5 participants were excluded from the study. There was a minor dropout during the data collection phase because of an early termination of the therapy due to participants’ health complications (*n* = 2), another minor attrition was present due to missing or incomplete post-treatment assessment (*n* = 3). Therefore, a total of 55 participants were finally involved in the study, divided into two groups. The control group consisted of 18 participants, all of whom were spa patients undergoing conventional spa therapy at the time of the study. The experimental group included 37 participants, who received VR therapy in addition to conventional spa treatment. Both groups had a similar age distribution, with averages of 65 and 61.3 years, respectively. The gender distribution was slightly imbalanced, particularly in the experimental group, where there were 11 men and 26 women, compared to the control group, which had 8 men and 10 women. The duration of the disease since its onset was also similar between the groups, with the experimental group averaging 9.41 years and the control group 9.08 years. Additionally, parameters related to participants’ physical activity were monitored, specifically occupation type (active/sedentary), engagement in sports (yes/no), and participation in sports during youth (yes/no). Demographic data for both groups is shown in Table 1.

### 3.1. Subjective Pain Assessment (VAS)

To evaluate the effectiveness of the methods used in addressing pain, the VAS was employed, recording values before and after the therapeutic intervention. Each point on the scale was scored on a 0–10 range, where the highest value indicates unbearable pain, and the lowest value indicates no pain. All baseline and post-treatment data for both groups are presented in Table 2. Although VAS represents an ordinal variable, normality testing of residuals and equality of variance in both groups were tested to consider using parametric one-way analysis of covariance (ANCOVA). Visual histogram analysis and Shapiro–Wilk test indicated normal distribution of residuals in both experimental and control group (*p* = 0.135 and 0.180, respectively) and Levene’s test indicated equal variances across groups (*p* = 0.819). Baseline differences were evaluated using Welch’s *t*-test, indicating equivalence with 90%CI fully contained in margin of equivalence (*p* = 0.3705, 90%CI [−0.7243; 0.7243]).

**Figure 1:**
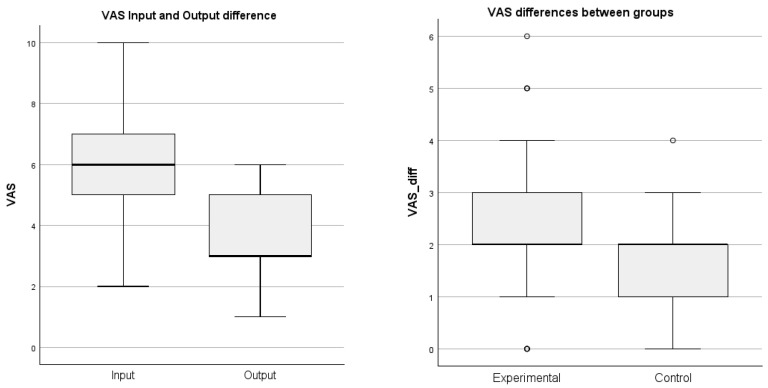
Difference in VAS Between Pre/Post Treatment and Between Groups. Circles represent values more than or below 1.5 × interquartile range (IQR).

To assess the overall effect of spa treatment, a comparison of the pre- and post-treatment VAS scores for all participants (*n* = 55), taking the group factor into account, was conducted using the parametric ANCOVA test. The results indicated a statistically significant difference (*p* < 0.001) between the mean pre- and post-treatment VAS scores, suggesting a positive impact of spa treatment on pain reduction with large effect size (*η*^2^ = 0.37). This difference is visualized in the boxplot on the left side of Figure 1. To assess the additive effect of VR on pain, the ANCOVA test suggested a statistically significant mean difference between groups (*p* = 0.045) with small to medium effect size (*η*^2^ = 0.08), which is visualized in the boxplot on the right side of Figure 1. Summary of detailed test results is presented in Table 3 and Table 4.

Given the considerable number of additional independent variables (specifically age, disease duration, gender, type of work, and physical activity), an analysis of interactions between the VAS parameter and these variables was conducted. Due to the ordinal nature of VAS and categorical factors, a nonparametric method was applied, specifically ordinal logistic regression. For the developed model, the Chi-square goodness-of-fit test indicated consistency between the model’s predicted and observed values (*p* = 0.632), and the test of parallel lines confirmed the absence of multicollinearity (*p* = 0.377).

**Figure 2:**
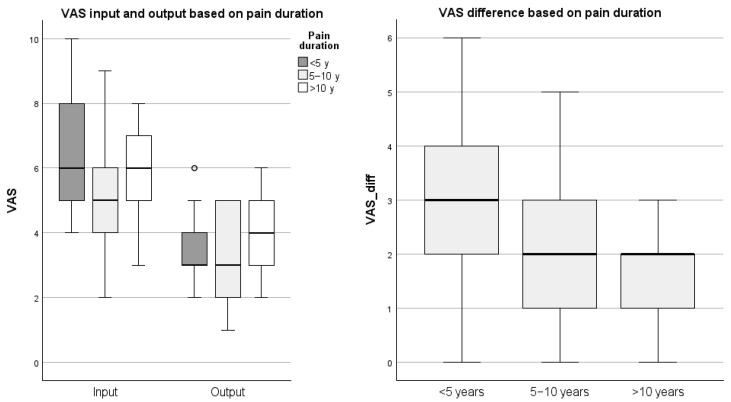
Relationship Between VAS Scores and Disease Duration of Participants. Circles represent values more than or below 1.5 × IQR.

Among the statistically significant results, a positive interaction was identified between the change in VAS and disease duration in the category of up to 5 years (*p* = 0.024), suggesting that a shorter disease duration is associated with greater changes in VAS scores (b = 1.620, 95%CI [0.21; 3.03]; SE = 0.72, Wald = 5.067). For the other parameters—gender (*p* = 0.778), age (*p* = 0.208), type of work (*p* = 0.751), physical activity (*p* = 0.212), and sports activity in youth (*p* = 0.312)—no statistically significant interactions were found. Boxplots of VAS scores and changes across the categories of disease duration are presented in Figure 2.

### 3.2. Range of Motion (VR)

To evaluate changes in range of motion, measured as time spent at specific distances from the centre of the shoulder joint (0.5, 0.6, 0.7, and 0.8 m), initial tests were conducted to verify the assumptions of the intended parametric tests. These included the Shapiro–Wilk test for normality and Mauchly’s test for sphericity. All baseline and post-treatment data with means and standard deviations are presented in Table 5.

The Shapiro–Wilk test indicated normal distribution for the differences in groups at 0.5, 0.6, and 0.7 m (*p* = 0.271, 0.052, and 0.051, respectively) but rejected normality for the differences at 0.8 m (*p* = 0.003). The results of Mauchly’s test rejected the null hypothesis (*p* < 0.001), indicating a violation of the sphericity assumption.

As a result, a two-way repeated measures ANOVA was ruled out as the primary method due to the substantial risk of results being affected by the violation of critical test assumptions. Since no nonparametric test fully met the requirements of our model, a paired *t*-test was used for each evaluated parameter at 0.5–0.7 m, and the Wilcoxon signed-rank test was applied for the evaluation of the parameter at 0.8 m.

**Figure 3:**
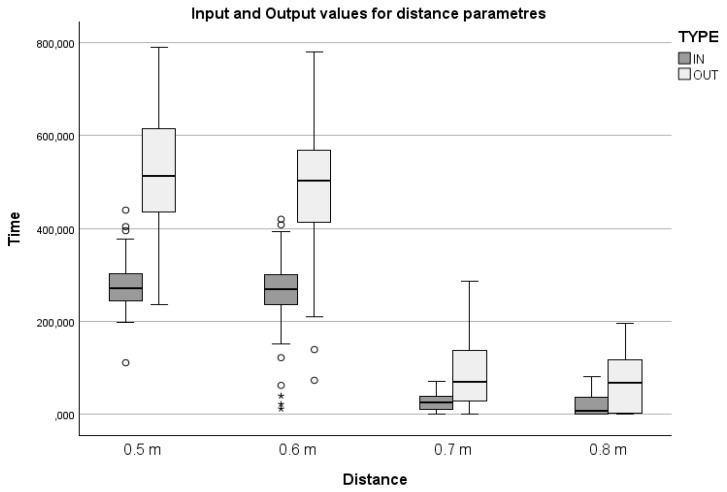
Differences Between Pre- and Post-Treatment Measurement Values for Distance. Circles represent values more than or below 1.5 × IQR, asterisks more than or below 3.0 × IQR.

Paired *t*-tests for parameters at 0.5, 0.6, and 0.7 m rejected the null hypothesis, indicating statistically significant differences between the mean pre- and post-treatment values (*p* < 0.001 for all, with medium to large effect sizes *d* = 1.63, 1.21, and 0.91, respectively). Similarly, the Wilcoxon signed-rank test also rejected the null hypothesis, demonstrating a statistically significant difference (*p* < 0.001 with large, adjusted effect size *d* = 0.91) between the medians of pre- and post-treatment values for the 0.8 m parameter. Boxplots displaying pre- and post-treatment values for all parameters are presented in Figure 3. Test results are summarized in Table 6.

To assess interactions between pre- and post-treatment differences across all distances and demographic variables (gender, age, disease duration, type of work, physical activity, and sports activity in youth), a one-way analysis of variance (ANOVA) was performed. Prior to conducting the test, the assumption of normality was checked using the Shapiro–Wilk test, which confirmed normality for all groups except the 5–10 years disease duration category (*p* = 0.019). Levene’s test for equality of variances did not reject the null hypothesis, indicating no statistically significant differences in variances between groups (*p* = 0.988).

Despite the violation of normality for one category, the parametric ANOVA method was applied due to its robustness against minor deviations from normality. However, the results did not reject the null hypothesis for any independent variable, revealing no statistically significant interactions between the dependent variable and the independent variables, including age (*p* = 0.953), gender (*p* = 0.368), disease duration (*p* = 0.370), type of work (*p* = 0.573), physical activity (*p* = 0.810), and sports activity in youth (*p* = 0.383), as well as their respective parameters.

## 4. Discussion

In our prospective, controlled observational study embedded in routine spa care, both groups showed reductions in pain and improvements in physical mobility, with more pronounced changes in the VR group. These findings suggest that VR, when added to comprehensive spa-based rehabilitation, may offer additional benefit as a complementary therapy.

The VAS results demonstrated a statistically significant reduction in pain levels among all participants, with a more notable decrease observed in the experimental group undergoing VR therapy. The Wilcoxon signed-rank test confirmed a significant reduction in median VAS scores from pre- to post-treatment (*p* < 0.001), supporting the overall effectiveness of traditional spa-based interventions. Furthermore, the Mann–Whitney U test identified a statistically significant difference between the experimental and control groups (*p* = 0.048), highlighting the additional benefit of VR therapy in enhancing pain management outcomes. Although this study was not designed to establish minimal clinically important differences, the magnitude of pain reduction observed, particularly in the VR group, falls within the range that is typically considered clinically meaningful in chronic musculoskeletal pain populations [30]. These results can be further explained through the distraction theory, which posits that immersive VR experiences engage cognitive and sensory pathways, effectively diverting attention away from nociceptive stimuli and reducing the subjective perception of pain [31]. VR also exerts anxiolytic effects, lowering stress and anxiety levels, which may further enhance pain relief and rehabilitation outcomes.

These findings are consistent with existing literature indicating that immersive VR experiences can modulate pain perception [32] by engaging cognitive and sensory pathways [33], thereby altering the individual’s focus from nociceptive stimuli. The interactive and stimulating design of the VR applications employed in this study likely facilitated these outcomes by redirecting attentional resources away from pain sensations and enhancing relaxation responses [34]. At the same time, some of the observed benefits may reflect nonspecific effects such as novelty, positive expectations or increased attention from staff, which cannot be clearly separated from specific neurophysiological mechanisms in this observational design. However, it is essential to consider that the balance of sensory stimulation in VR must be carefully managed, as excessive stimuli or overly complex combinations may have a disruptive effect, leading to a loss of concentration and reduced motivation, potentially compromising the therapeutic effectiveness [13].

Compared to traditional physiotherapy and biofeedback, VR therapy may offer additional benefits by combining physical activity with cognitive engagement in a multisensory environment [35]. This immersive nature of VR not only enhances patient motivation but may also explain the more pronounced improvements in pain reduction and mobility observed in our study.

Building on these results, the ordinal logistic regression analysis revealed a statistically significant association between pain reduction [17] and disease duration [36,37], with the most pronounced effects observed in patients with a disease history of up to five years (*p* = 0.024). This finding suggests that VR interventions may yield greater therapeutic benefits for individuals in the earlier stages of chronic musculoskeletal and neurogenic conditions, potentially due to enhanced neuroplasticity and a heightened responsiveness to novel rehabilitative stimuli [38] within this patient cohort.

The evaluation of physical mobility further demonstrated significant improvements in the experimental group, particularly in the range of motion across multiple distances from the shoulder joint (0.5, 0.6, 0.7, and 0.8 m). Both paired *t*-tests and Wilcoxon signed-rank tests confirmed these enhancements as statistically significant (*p* < 0.001 for all measured distances), indicating the effectiveness of VR-enhanced rehabilitation protocols in promoting motor function. The observed improvements in mobility suggest that VR-based therapeutic activities enhance motor function through structured, repetitive, and task-specific exercises. Repetitive movements, such as those performed during virtual fruit picking and constellation recognition, are known to reinforce motor learning by facilitating neural plasticity and strengthening neuromuscular pathways [4]. This consistent engagement with purposeful tasks likely contributed to improved coordination, flexibility, and overall functional mobility [39], highlighting the potential of VR as an effective modality in rehabilitation programs. Interestingly, despite significant gains in mobility, the ANOVA analysis did not reveal any statistically significant interactions between mobility improvements and demographic variables such as age, gender, disease duration, type of work, or physical activity levels. This suggests that VR therapy’s benefits on physical mobility are broadly applicable across diverse patient demographics, making it a versatile tool in rehabilitation.

While the study offers valuable insights, certain limitations should be noted. The sample size, particularly in the control group, was small but consistent with the scope of a small-scale experimental study [40], and the groups were numerically imbalanced, which limits the precision of the effect estimates. Patients were allocated to VR or standard care according to their preferences and treatment availability, and blinding of participants and clinical staff, including outcome assessors, was not feasible in the context of routine spa care. This nonrandomized, preference-based allocation and lack of blinding introduce a risk of selection bias and expectancy or Hawthorne effects, including placebo and novelty responses. The three-week intervention period limits conclusions about the long-term effects of VR therapy on pain and mobility, and no follow-up data are available on the durability of changes. Future research should include larger, more diverse populations and extended follow-ups to assess the sustainability of VR’s benefits. Overall, our findings should be regarded as preliminary and hypothesis-generating rather than confirmatory. Larger randomized studies with more diverse and balanced samples, standardized VR protocols and longer follow-up are needed to determine whether the observed benefits of VR are robust and clinically meaningful. Further exploration of the mechanisms behind VR-induced pain relief and mobility improvements, along with patient adherence and satisfaction, will enhance its application in rehabilitation.

## 5. Conclusions

This prospective, controlled observational study embedded in routine spa care suggests that integrating VR into comprehensive spa treatment may be associated with reduced pain and improved physical mobility in patients with chronic musculoskeletal and neurogenic conditions. In our sample, VR-enhanced rehabilitation was associated with additional improvements beyond conventional therapies, particularly in pain outcomes and measures of motor function. The most pronounced pain reduction was observed in patients with shorter disease duration, which may indicate a greater responsiveness to VR-based interventions in earlier stages of chronic conditions. This observation remains exploratory. Given the nonrandomized allocation, limited sample size and short follow-up, these results should be regarded as preliminary and hypothesis-generating rather than definitive evidence of efficacy. VR can be considered a promising complementary tool in spa-based rehabilitation. Its effectiveness and optimal implementation, however, need to be confirmed in randomized controlled trials with larger, balanced samples, standardized VR protocols and long-term follow-up.

## Figures and Tables

**Table 1:** Demographic Data and Characteristics for Both Groups.

Group	Age	Disease Duration	Sex		Occupation		Sports		Sports in Youth	
			Male	Female	Active	Sedentary	Yes	No	Yes	No
Label (*n*)	Mean (SD)	Mean (SD)	*n*		*n*		*n*		*n*	
EXP (*n* = 37)	65 (11.39)	9.41 (9.08)	11	26	19	18	21	16	31	6
CON (*n* = 18)	61.3 (12.49)	9.08 (9.77)	8	10	10	8	8	10	10	8

**Table 2:** Baseline and Post-Treatment Data for Both Groups in VAS Variable.

VAS	Baseline		Post-Treatment	
Label (*n*)	Mean (SD)	Median	Mean (SD)	Median
EXP (*n* = 37)	6 (1.8)	6	3.46 (1.37)	3
CON (*n* = 18)	5.61 (1.33)	6	3.89 (1.49)	4
Difference	0.39	0	−0.43	1

**Table 3:** Results of ANCOVA Test with Groups and Pre/Post-Treatment Effects.

VAS	Mean Square	*F*	*p*	Effect Size
(*n* = 55)				*η* ^2^
Group	5.11	4.21	0.045	0.08
Baseline	37.66	30.99	<0.001	0.37

**Table 4:** Results of ANCOVA Test with Mean Difference Between Groups.

VAS	Difference	95%CI		*p*	Effect Size
(*n* = 55)	Mean (SE)	Lower	Upper		*η* ^2^
Group	0.65 (0.32)	0.014	1.29	0.045	0.08

**Table 5:** Baseline and Post-Treatment Data for Both Groups in Range of Motion Variable.

RoM	Baseline		Post-Treatment		Difference	
(*n* = 37)	Mean (SD)	Median	Mean (SD)	Median	Mean (SD)	Median
0.5 m	280.76 (61.80)	271.97	514.25 (140.45)	513.22	233.49 (143.09)	237.34
0.6 m	253.63 (96.08)	268.77	492.26 (160.26)	503.78	238.62 (197.77)	235.59
0.7 m	24.75 (18.19)	25.83	87.7 (70.66)	71.22	62.94 (69.48)	43.81
0.8 m	21.49 (24.41)	7.93	68.44 (65.01)	67.64	46.95 (51.75)	32.64

**Table 6:** Summary of Test Results in Baseline and Post-Treatment Comparison.

RoM	Difference	95%CI		*p*	Effect Size	95%CI	
(*n* = 37)	Mean (SD)	Lower	Upper		Cohen’s *d*	Lower	Upper
0.5 m	233.49 (143.09)	185.78	281.20	<0.001	1.63	1.13	2.12
0.6 m	238.62 (197.77)	304.57	172.68	<0.001	1.21	0.78	1.63
0.7 m	62.94 (69.48)	39.78	86.11	<0.001	0.91	0.52	1.29
0.8 m	46.95 (51.75)	29.70	64.20	<0.001	0.91	0.52	1.29

## Data Availability

Data available upon reasonable request from the corresponding author.

## Abbreviations

The following abbreviations are used in this manuscript:

ANOVA	Analysis of variance
ANCOVA	Analysis of covariance
IQR	Interquartile range
VAS	Visual analog scale
VR	Virtual reality

## References

[1] Lochmannová A. (2025). Exploring the role of virtual reality in preparing emergency responders for mass casualty incidents. Isr. J. Health Policy Res..

[2] Bednář M., Dufek P., Lochmannová A., Šimon M., Bureš M. (2024). Use of virtual reality for education and training of emergency rescue system for crisis situations. Proceedings of the 15th International Conference on Education Technology and Computers (ICETC ’23).

[3] Pniak B., Leszczak J., Kurczab J., Krzemińska A., Pięta J., Plis A., Czenczek-Lewandowska E., Guzik A. (2021). The efficiency of spa rehabilitation in chronic ischemic stroke patients—Preliminary reports. Brain Sci..

[4] Zhang B., Li D., Liu Y., Wang J., Xiao Q. (2021). Virtual reality for limb motor function, balance, gait, cognition and daily function of stroke patients: A systematic review and meta-analysis. J. Adv. Nurs..

[5] Feitosa J.A., Fernandes C.A., Casseb R.F., Castellano G. (2022). Effects of virtual reality based motor rehabilitation: A systematic review of fMRI studies. J. Neural Eng..

[6] Fang Z., Wu T., Lv M., Chen M., Zeng Z., Qian J., Chen W., Jiang S., Zhang J. (2022). Effect of traditional plus virtual reality rehabilitation on prognosis of stroke survivors: A systematic review and meta-analysis of randomized controlled trials. Am. J. Phys. Med. Rehabil..

[7] Blyth F.M., Noguchi N. (2017). Chronic musculoskeletal pain and its impact on older people. Best Pract. Res. Clin. Rheumatol..

[8] Adriaansen J.J.E., Ruijs L.E.M., van Koppenhagen C.F., van Asbeck F.W.A., Snoek G.J., van Kuppevelt D., Visser Meily J.M.A., Post M.W.M. (2016). Secondary health conditions and quality of life in persons living with spinal cord injury for at least ten years. J. Rehabil. Med..

[9] Gonzalez-Sanchez M., Reina-Ruiz Á.J., Molina-Torres G., Trzcińska S.K., Carrasco-Vega E., Lochmannová A., Galán-Mercant A. (2025). Structural and Psychometric Properties of Neck Pain Questionnaires Through Patient-Reported Outcome Measures: A Systematic Review. Medicina.

[10] Bergman S. (2007). Management of musculoskeletal pain. Best Pract. Res. Clin. Rheumatol..

[11] Sramka M., Lacko J., Ruzický E., Masan J. (2020). Combined methods of rehabilitation of patients after stroke: Virtual reality and traditional approach. Neuroendocrinol. Lett..

[12] Zhang C., Yu S. (2024). Technology to enhance patient motivation in virtual reality rehabilitation: A review. Games Health J..

[13] Kubr J., Lochmannová A., Hořejší P. (2024). Immersive virtual reality training in industrial settings: Effects on memory retention and learning outcomes. IEEE Access.

[14] Maggio M.G., Russo M., Foti Cuzzola M., Destro M., La Rosa G., Molonia F., Bramanti P., Lombardo G., De Luca R., Calabrò R.S. (2019). Virtual reality in multiple sclerosis rehabilitation: A review on cognitive and motor outcomes. J. Clin. Neurosci..

[15] Wittkopf P.G., Lloyd D.M., Coe O., Yacoobali S., Billington J. (2020). The effect of interactive virtual reality on pain perception: A systematic review of clinical studies. Disabil. Rehabil..

[16] Pourmand A., Davis S., Marchak A., Whiteside T., Sikka N. (2018). Virtual reality as a clinical tool for pain management. Curr. Pain Headache Rep..

[17] Alemanno F., Houdayer E., Emedoli D., Locatelli M., Mortini P., Mandelli C., Raggi A., Iannaccone S. (2019). Efficacy of virtual reality to reduce chronic low back pain: Proof-of-concept of a non-pharmacological approach on pain, quality of life, neuropsychological and functional outcome. PLoS ONE.

[18] Teo W.P., Muthalib M., Yamin S., Hendy A.M., Bramstedt K., Kotsopoulos E., Perrey S., Ayaz H. (2016). Does a combination of virtual reality, neuromodulation and neuroimaging provide a comprehensive platform for neurorehabilitation?—A narrative review of the literature. Front. Hum. Neurosci..

[19] Tack C. (2021). Virtual reality and chronic low back pain. Disabil. Rehabil. Assist. Technol..

[20] Canning C.G., Allen N.E., Nackaerts E., Paul S.S., Nieuwboer A., Gilat M. (2020). Virtual reality in research and rehabilitation of gait and balance in Parkinson disease. Nat. Rev. Neurol..

[21] Huseynli A., Marková V., Špišák L. (2025). Preliminary Study: The Potential Role of a One-Week Comprehensive Spa Treatment on Mental Health Prevention of Adult Patients in Konstantinovy Lázně Spa, Czech Republic. Int. J. Spa Wellness.

[22] Chow S.-C., Shao J., Wang H., Lokhnygina Y. (2017). Sample Size Calculations in Clinical Research.

[23] Lan X., Li L., Jia Q., He F., Kuang G., Zeng W., Chen M., Guo C., Wen Z., Chen Q. (2025). Physical modalities for the treatment of knee osteoarthritis: A network meta-analysis. Aging Clin. Exp. Res..

[24] Bjerre-Bastos J., Li Y., Andersen J., Christiansen C., Karsdal M., Bihlet A. (2021). The association between single-question vas pain rating and weight-bearing and non-weightbearing domains of womac pain: A post-hoc analysis based on data from two phase 3 clinical trials. Osteoarthr. Cartil..

[25] Lee Y., Lee S., Lee Y.J., Ha I. (2023). Minimum clinically important difference for nonsurgical interventions for spinal diseases: Choosing the appropriate values for an integrative medical approach. Perspect. Integr. Med..

[26] Carlsson A.M. (1983). Assessment of chronic pain. I. Aspects of the reliability and validity of the visual analogue scale. Pain.

[27] Hawker G.A., Mian S., Kendzerska T., French M. (2011). Measures of adult pain: Visual Analog Scale for Pain (VAS Pain), Numeric Rating Scale for Pain (NRS Pain), McGill Pain Questionnaire (MPQ), Short-Form McGill Pain Questionnaire (SF-MPQ), Chronic Pain Grade Scale (CPGS), Short Form-36 Bodily Pain Scale (SF-36 BPS), and Measure of Intermittent and Constant Osteoarthritis Pain (ICOAP). Arthritis Care Res..

[28] Goulet-Pelletier J., Cousineau D. (2018). A review of effect sizes and their confidence intervals, Part I: The Cohen’s d family. Quant. Methods Psychol..

[29] Levine T.R., Hullett C.R. (2002). Eta squared, partial eta squared, and misreporting of effect size in communication research. Hum. Commun. Res..

[30] Salaffi F., Stancati A., Silvestri C.A., Ciapetti A., Grassi W. (2004). Minimal Clinically Important Changes in Chronic Musculoskeletal Pain Intensity Measured on a Numerical Rating Scale. Eur. J. Pain.

[31] Gupta A., Scott K., Dukewich M. (2018). Innovative technology using virtual reality in the treatment of pain: Does it reduce pain via distraction, or is there more to it?. Pain Med..

[32] Ang S.P., Montuori M., Trimba Y., Maldari N., Patel D., Chen Q.C. (2021). Recent applications of virtual reality for the management of pain in burn and pediatric patients. Curr. Pain Headache Rep..

[33] Barcatta K., Holl E., Battistutta L., van der Meulen M., Rischer K.M. (2022). When less is more: Investigating factors influencing the distraction effect of virtual reality from pain. Front. Pain Res..

[34] Brown P., Powell W., Dansey N., Al-Abbadey M., Stevens B., Powell V. (2022). Virtual reality as a pain distraction modality for experimentally induced pain in a chronic pain population: An exploratory study. Cyberpsychol. Behav. Soc. Netw..

[35] Parisi A., Bellinzona F., Di Lernia D., Repetto C., De Gaspari S., Brizzi G., Riva G., Tuena C. (2022). Efficacy of multisensory technology in post-stroke cognitive rehabilitation: A systematic review. J. Clin. Med..

[36] Anderson J.J., Wells G., Verhoeven A.C., Felson D.T. (2000). Factors predicting response to treatment in rheumatoid arthritis: The importance of disease duration. Arthritis Rheum..

[37] Kubricht V., Ševčík P. (2017). Chronic postsurgical pain in mixed surgical population: Does an acute pain service make a difference?. Bratisl. Lek. Listy.

[38] Bohil C.J., Alicea B., Biocca F.A. (2011). Virtual reality in neuroscience research and therapy. Nat. Rev. Neurosci..

[39] Mao Y., Chen P., Li L., Huang D. (2014). Virtual reality training improves balance function. Neural Regen. Res..

[40] Cash P., Elias E., Dekoninck E., Culley S. (2012). Methodological insights from a rigorous small scale design experiment. Des. Stud..

